# Magnetic Sensor for Building Structural Vibrations

**DOI:** 10.3390/s140202468

**Published:** 2014-02-05

**Authors:** Alfonso García, Carlos Morón, Enrique Tremps

**Affiliations:** Grupo de Sensores y Actuadores, Departamento de Tecnología de la Edificación, Universidad Politécnica de Madrid, 28040 Madrid, Spain; E-Mails: alfonso.garciag@upm.es (A.G.); enrique.tremps@upm.es (E.T.)

**Keywords:** magnetic sensor, displacement, vibration, self-induction

## Abstract

This paper shows a new displacement-to-frequency transducer based on the variation of a coil inductance when a magnetic core is partially or completely inserted inside. This transducer is based on a Colpitts oscillator due its low manufacturing price, behavior and immunity to noise. A tank circuit with a configuration in parallel was used because it can be employed at lower frequencies and it enables it to make a direct analysis. The sensor has a dynamic range equal to the length of the coil. The cores can exchange sensors (coils with its ferromagnetic core) using the same electronic measuring system. In this way, with only an electronic circuit, the core sensor determines the measurement range. The obtained resolution is higher than 1/100,000, and the sensor also allows the measurement and knowing in real time the effect of vibration, thermal expansion, referred overload movements, *etc.*., that can occur in the structural elements of a building.

## 1. Introduction

To ensure the reliable evaluation of a building structure's condition and to know its stability conditions displacement measurements are required. There are many devices to measure these displacements: capacitive, accelerometer, optical and inductive sensors and real-time kinematic global positioning systems [1–2].

Inductive Linear Variable Differential Transformer (LVDT) sensors are some of the most used sensors in displacement measurements due to the advantages offered by their electrical insulation between the sensor and measurement electronics; their hardness that allows them to be used in unclean or unfavorable environments and their reliability that allows them to make measurements with a higher accuracy than 1/10,000 [3–7].

But, they some disadvantages such as their dimensions (the range a LVDT measures is always less than ½ of the total length of the windings) and the complexity of the associated measurement electronics (voltage and phase) that need to be detected in order to discriminate the direction and magnitude of the displacement and the fact a good stabilization of the working frequency is required. As they are mutual induction amplitude systems, they depend on the frequency of the driving signal [8–11].

In this work a new displacement-to-frequency transducer based on the variation of the self-inductance of a coil is shown, when the magnetic core (sensor nucleus) is partially or completely inserted in a single coil. This transducer is based in a Colpitts oscillator circuit that has low manufacturing prices, and a great immunity to noise. A tank circuit with a parallel configuration was used because it can be used at lower frequencies and allows making a direct analysis.

The sensor was designed using four coils of different sizes and also with a different number of turns but with the same self-inductance (L = 39.5 μH). In this way, the electronic circuit can be the same for different sensor cores. Thus, as the sensor has a dynamic range equal to the length of the inductance, the core sensor (coil with its ferromagnetic core) can be exchanged with the same electronic measuring system. In this way, the range of measurement is only determined by the core sensor selected, using the same electronics for all sensor cores. The resolution obtained is higher than 1/100,000.

## 2. The Transducer

**Figure 1.**
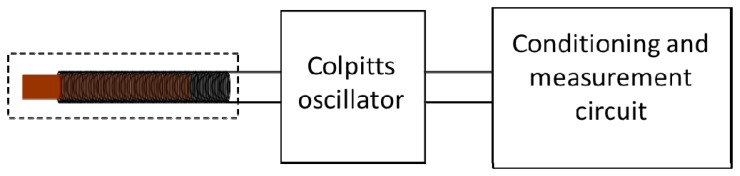
Block diagram of the transducer.

Figure 1 shows the block diagram of the magnetic transducer. The sensor head is connected with the circuit oscillator whose frequency of oscillation depends on the self-induction of the sensor head made of a coil and a sliding ferromagnetic core. The next is the electronic signal conditioning and measurement block. Essentially it takes the signal of the oscillator, fits the amplitude and measures the frequency of the same one correlating it with the corresponding distance.

### 2.1. The Electronic Circuit

**Figure 2.**
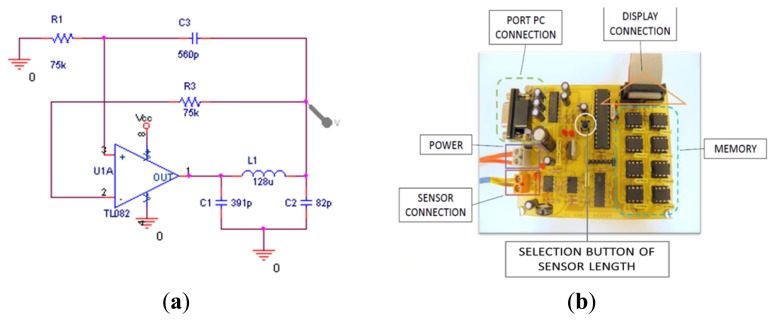
(**a**) General scheme of a Colpitts oscillator operating as an active element; (**b**) Developed electronic sensor.

The general structure of a standard Colpitts oscillator with an operational amplifier as an active element is shown in Figure 2, where C1 and C2 are capacitors and L1 the coil. The negative feedback circuit will oscillate if the transfer function loop reaches a unit amplitude (0 dB) when the phase shift is −180°.

### 2.2. Coil Design and Construction

It was decided that coils of different longitude would be developed, but with the same self-inductance. In this way, the dynamic range of the transducer (coil length) can be chosen without the need to make adjustments (or changes) in the electronic circuitry. The self-induction of a coil without a ferromagnetic core can be obtained as:

(1) $$L=\mu_{0}\cdot A\cdot {\left(\frac{N}{l}\right)}^{2}\cdot l=\mu_{0}\cdot A\cdot \frac{N^{2}}{l}$$

where *μ_0_* is the air magnetic permeability, A is the cross sectional area of coil, N is the number of turns and *l* the total length of coil.

Taking into account the section of wire *Φ* (for the same winding base), the total length is:

(2) l=Nϕ

The cross sectional area of the coil (taking into account that it is only one layer of coil winding) must change with the wire section *Φ*:

(3) $$A={\left(d+\phi \right)}^{2}\pi$$

where *d* is the tubular base diameter of winding. Thus, Equation (1) becomes:

(4) $$L=\mu_{0}\cdot \frac{N}{\phi }\cdot {\left(d+\phi \right)}^{2}\cdot \pi =\mu_{0}\cdot \pi \cdot N\left(\frac{d^{2}}{\phi }+2d+\phi \right)$$

To ensure that two different coils (with different wire sections and different total length) have the same self-induction, it is necessary that the relation between the numbers of winding be:

(5) $$N\left(\frac{d^{2}}{\phi }+2d+\phi \right)=N^{\prime }\left(\frac{d^{2}}{\phi^{\prime }}+2d+\phi^{\prime }\right)$$

Four different coils have been made. Table 1 shows the coils that have been developed: their length, theoretical number and the real number of turns.

**Figure 3.**
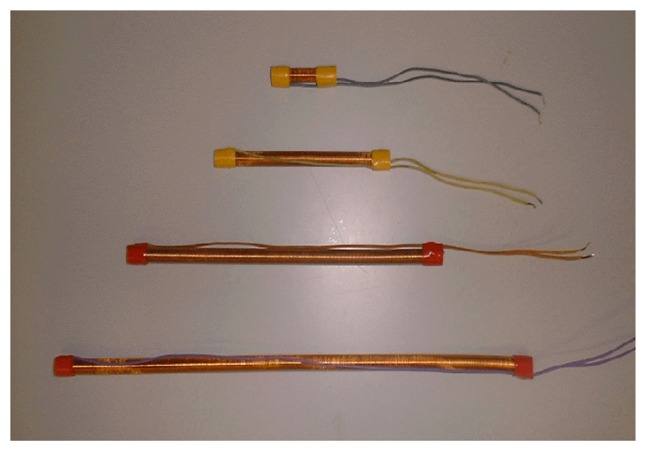
Different developed coils.

Figure 3 shows the built coils. All coils are wound over the same tubular base. There is only one layer of wound coil; different enameled copper wire has been used and different coil lengths have been obtained, but all coils have the same self-induction: 39.5 μH.

## 3. Properties of the Transducer

**Figure 4.**
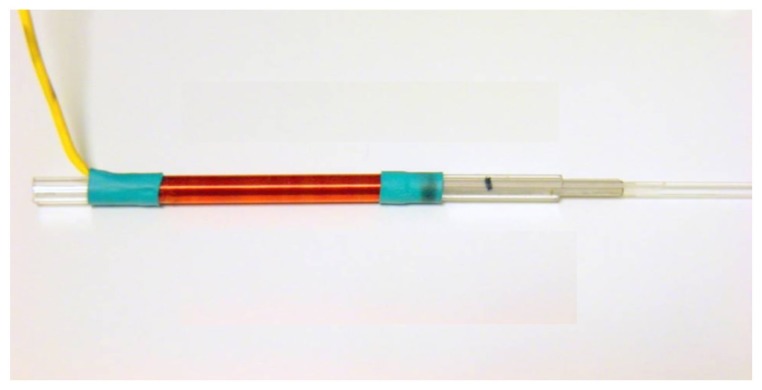
Sliding ferromagnetic core.

Two magnetic materials with different properties have been used as sliding ferromagnetic cores (Figure 4) to see how the transducer behaves with each of them. In one case a 3 mm diameter mild steel bar was used and in another case six sheets of an electrically isolated amorphous magnetic material (METGLAS 2705M, Metglas^®^, Inc., Conway, SC, USA) was used.

To obtain the range of the transducer, with both materials used for the nucleus, the frequencies with only the coil (without magnetic material inside) and when all magnetic nuclei are inserted inside them have been measured. The procedure was repeated for each of the four built coils. In Table 2 the frequency range (maximum frequency range of sensor response) and the frequency obtained for the four coils are shown.

### 3.1. Calibration

**Figure 5.**
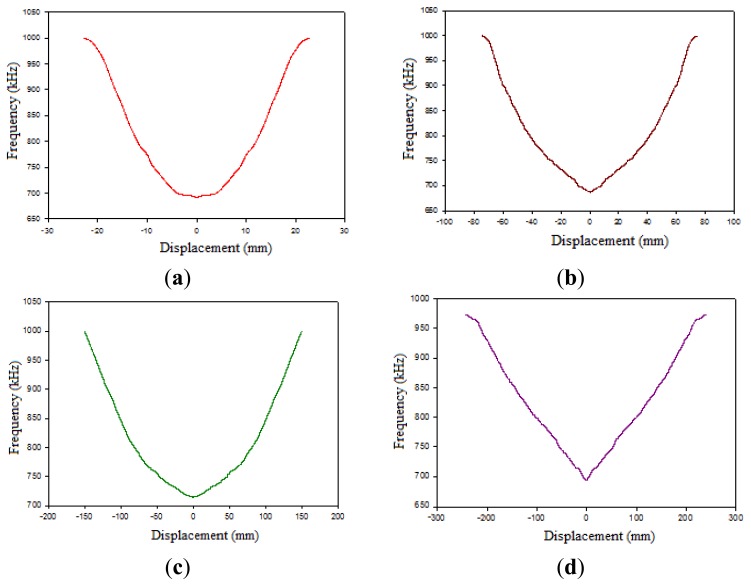
Calibration curve of coils with metallic glass as core and different length: (**a**) 19.3 mm; (**b**) 92.7 mm; (**c**) 178 mm; (**d**) 280 mm.

Calibrations for the transducer have been made with all the coils and mild steel and METGLASS 2705M materials as magnetic nucleus. Figure 5 shows the results obtained for one tested coil with a mild steel bar or METGLASS 2705M as sensor core.

### 3.2. Resolution and Threshold

The threshold at 0 mm has been obtained and this parameter can be expressed as signal/noise ratio:

(6) $$\frac{S}{N}=20\log\left(\frac{e_{1}}{e_{0}}\right)\left(\;dB\right)$$

In Table 3 the transducer thresholds for the four developed coils are shown.

### 3.3. Thermal Stability

Studies of the thermal stability of the transducer have been performed. To do this, a heater has been used and the frequency of response for different displacements has been measured by varying the temperature (the displacement was maintained constant for each temperature variation). The thermal study has been done with a muffle furnace with a temperature control that provides a precision of ±1 °C for three temperatures (20 °C, 40 °C and 60 °C). It has been observed that the maximum frequency variation was 24 Hz—equivalent to 21 μm (below the sensor accuracy 33.5 Hz—equivalent to 28 μm).

In this way, the frequency response variation with temperature obtained was almost negligible.

### 3.4. Results

**Figure 6.**
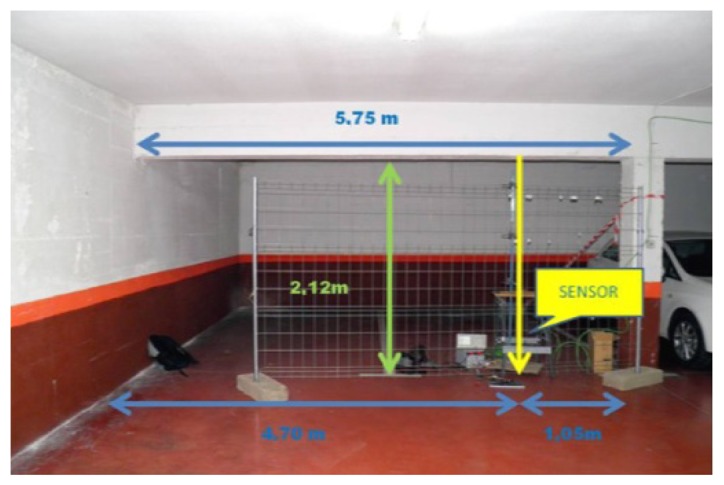
Schematic sensor positioning at the parking garage.

The sensor with 280 mm length coil was placed at 1.05 m from the column (Figure 6) to measure the vibration produced by the passage of cars in an underground parking garage.

**Figure 7.**
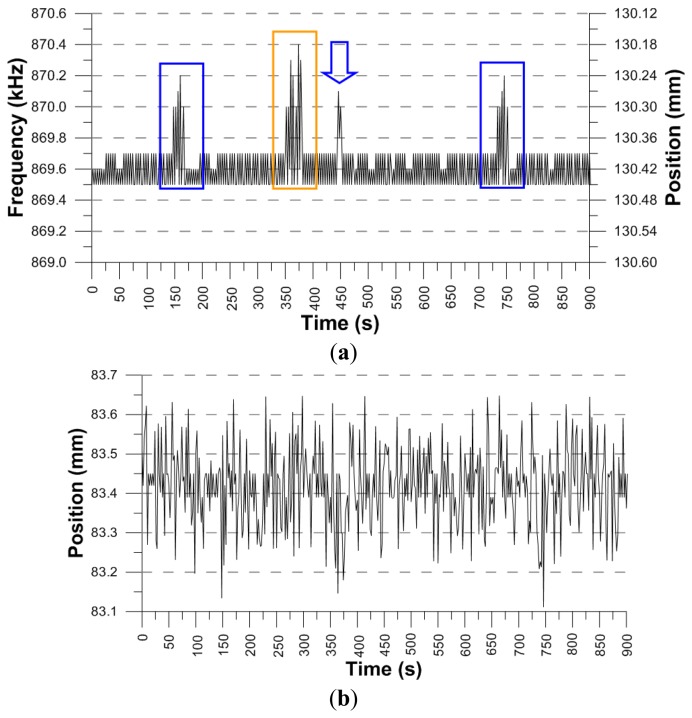
(**a**) Sensor response with a 280 mm length coil; (**b**) LVDT response under the same conditions.

In Figure 7 the 280 mm length coil response can be seen. The data has been updated every 2 s. The areas marked with a box correspond to the parking garage. The marks in orange correspond to cars leaving the low floor parking garage and the marks in blue, to cars going out of upper floor parking garage. Within these areas the highest peak coincides with the closure of the parking door. Also, the areas marked with an arrow indicate traffic on the road outside just above the underground parking garage. The other relevant peaks, between 618 and 618.1 kHz for example, probably indicate the exits or entries from the street parking lot.

## 4. Conclusions

A displacement-to-frequency transducer has been built, whose dynamic range can be changed simply by exchanging a sensor head for another, without making any adjustments or changes to the associated electronic devices. This transducer is based on a single coil (LVDTs need three coils). The dynamic range is nearly equal to the length of the coil (LVDTs have a dynamic range of less than ½ of the total length of windings). The device shows a high thermal stability. As it is a magnetic device, there is electrical insulation between the sensor head and the associated electronics. Furthermore, the device is not very expensive. All these features make this transducer suitable for all types of industrial applications, including those in hazardous locations or large contaminated areas.

## Figures and Tables

**Table 1.** Characteristic of the different coils.

**Wire Diameter (mm)**	**Length (mm)**	**Theoretical Winding Number**	**Actual Winding Number**
0.400	280	700	690
0.315	178	566	555
0.224	92.7	414	405
0.100	19.3	193	181

**Table 2.** Transducer thresholds detection for different coils.

Coil Length (mm)	Magnetic Material	Range (mm)	Frequency Range (kHz)	Precision (Hz/μm)
19.3	Steel	19	102	5
	Metglas	19	313	16
92.7	Steel	93	96	1
	Metglas	93	308	3
178	Steel	178	100	0.5
	Metglas	178	305	1.7
280	Steel	280	95	0.3
	Metglas	280	335	1

**Table 3.** Transducer thresholds for different coils.

**Coil Length (mm)**	**Mild Steel Bar (dB)**	**METGLASS 2705M (dB)**
19.3	2.07	3.21
92.7	1.76	3.25
178	0.98	2.92
280	0.91	3.19
